# Supplementary material for: Skin aging risk factors: A nationwide population study in Mongolia risk factors of skin aging
Source: PLoS One. 2022 Jan 19;17(1):e0249506. doi: 10.1371/journal.pone.0249506 (PMC8769290; doi:10.1371/journal.pone.0249506)
Supplement: S1 File — (DOCX) [file pone.0249506.s001.docx]

**QUESTIONNAIRE AND CLINICAL EXAMINATION FORM**

(All questions are single response unless indicated otherwise)

| 1. **Personal details** | | | | | | | | | | | | | | | | | | |
| --- | --- | --- | --- | --- | --- | --- | --- | --- | --- | --- | --- | --- | --- | --- | --- | --- | --- | --- |
|  | Codes | Question | | | | | | Answer | | | | | | | | | | Codes |
| 1 | I1 | Address | | | | | |  | | | | | | | | | | |
| 2 | I2 | Questionnaire obtained date | | | | | | /_ _ / _ _ _ / _ _ _ _ /  dd mmm yyyy | | | | | | | | | | |
| 3 | I3 | Tel/ mobile | | | | | |  | | | | | | | | | | |
| 1. **Demographic questions** | | | | | | | | | | | | | | | | | | |
| 4 | De1 | Sex | | | | | | Male | | | | | | | | | | 1 |
| 5 | De2 |  |  |  |  |  |  | Female | | | | | | | | | | 2 |
| 6 | De3 | Birth Date | | | | | | /_ _ / _ _ _ / _ _ _ _ /  dd mmm yyyy | | | | | | | | | | |
| 7 | De4 | Age | | | | | | _ _ _ years_ _ month | | | | | | | | | | |
| 8 | De5 | How many members are in your family? | | | | | |  | | | | | | | | | | |
| 9 | De6 | What is your marital status? | | | | | | Married | | | | | | | | | | 1 |
| 10 | De7 |  |  |  |  |  |  | Divorced | | | | | | | | | | 2 |
| 11 | De8 |  |  |  |  |  |  | Never married | | | | | | | | | | 3 |
| 12 | Di9 | What is your highest level of education? | | | | | | High school | | | | | | | | | | 1 |
|  |  |  |  |  |  |  |  | College | | | | | | | | | | 2 |
|  |  |  |  |  |  |  |  | Bachelors | | | | | | | | | | 3 |
|  |  |  |  |  |  |  |  | Postgraduate | | | | | | | | | | 4 |
| 13 | SE2 | Which best describes your working conditions? | | | | | | Nightshift | | | | | | | | | | 1 |
|  |  |  |  |  |  |  |  | Outside | | | | | | | | | | 2 |
|  |  |  |  |  |  |  |  | Heavy | | | | | | | | | | 3 |
|  |  |  |  |  |  |  |  | Hazardous (mineral and coal extraction, etc.) | | | | | | | | | | 4 |
|  |  |  |  |  |  |  |  | Optimal | | | | | | | | | | 5 |
| 15 | SE3 | Where do you live? | | | | | | City | | | | | | | | | | 1 |
|  |  |  |  |  |  |  |  | Aimag (province) | | | | | | | | | | 2 |
|  |  |  |  |  |  |  |  | Sum (village) | | | | | | | | | | 3 |
|  |  |  |  |  |  |  |  | Countryside | | | | | | | | | | 4 |
|  |  |  |  |  |  |  |  | Other | | | | | | | | | | 5 |
| 16 | SE4 | How many years have you lived in the place you currently live? | | | | | | | _ _ _ years | | | | | | | | |  |
| 17 | SE5 | What type of home does your household live in? | | | | | | | Ger | | | | | | | | | 1 |
|  |  |  |  |  |  |  |  |  | Ger district house | | | | | | | | | 2 |
|  |  |  |  |  |  |  |  |  | Dormitory | | | | | | | | | 3 |
|  |  |  |  |  |  |  |  |  | Apartment | | | | | | | | | 4 |
|  |  |  |  |  |  |  |  |  | Other | | | | | | | | | 5 |
| 18 | SE6 | What type of water supply do you have at your home? | | | | | | | Central water system, with hot and cold water | | | | | | | | | 1 |
|  |  |  |  |  |  |  |  |  | Central water system, with cold water only | | | | | | | | | 2 |
|  |  |  |  |  |  |  |  |  | Water distribution point connected to the central system | | | | | | | | | 3 |
|  |  |  |  |  |  |  |  |  | Water supply track | | | | | | | | | 4 |
|  |  |  |  |  |  |  |  |  | Deep well water (jet pump system) | | | | | | | | | 5 |
|  |  |  |  |  |  |  |  |  | Hand-pumped well water | | | | | | | | | 6 |
|  |  |  |  |  |  |  |  |  | Springwater | | | | | | | | | 7 |
|  |  |  |  |  |  |  |  |  | River water | | | | | | | | | 8 |
|  |  |  |  |  |  |  |  |  | Snow and ice water | | | | | | | | | 9 |
|  |  |  |  |  |  |  |  |  | Other | | | | | | | | | 10 |
| 19 | SE7 | What is your household's monthly income (MNT)? | | | | | | |  | | | | | | | | | |
| 1. **Lifestyles questions** | | | | | | | | | | | | | | | | | | |
| 20 | SE8 | What kind of food do you usually eat? | | | | | | | Meat dishes | | | | | | | | | 1 |
|  |  |  |  |  |  |  |  |  | Vegetarian dishes | | | | | | | | | 2 |
| 21 | SE9 | If vegetarian, how long have you been on a mainly vegetarian diet? | | | | | | | Up to one year | | | | | | | | | 1 |
|  |  |  |  |  |  |  |  |  | 1-4 years | | | | | | | | | 2 |
|  |  |  |  |  |  |  |  |  | 5-9 years | | | | | | | | | 3 |
|  |  |  |  |  |  |  |  |  | 10 years or more | | | | | | | | | 4 |
| 22 | GF1 | How much time do you sleep at night? | | | | | | | 4-5 hours | | | | | | | | | 1 |
|  |  |  |  |  |  |  |  |  | 6-7 hours | | | | | | | | | 2 |
|  |  |  |  |  |  |  |  |  | 8-10 hours | | | | | | | | | 3 |
| 23 | GF2 | In the summer, approximately how much time do you spend in the sun from 7 am to 6 pm? | | | | | | | .......... hours | | | | | | | | | |
|  |  |  |  |  |  |  |  |  | ........ minutes | | | | | | | | | |
| **3.1 Tobacco consumption** | | | | | | | | | | | | | | | | | | |
| 24 | GF6 | Do you currently use any tobacco? | | | | | | | Yes | | | | | | | | 1 | |
|  |  |  |  |  |  |  |  |  | No | | | | | | | | 2 | |
| 25 | GF7 | At what age did you start smoking? | | | | | | | _ _ years | | | | | | | |  | |
|  |  |  |  |  |  |  |  |  | Don't know | | | | | | | | 99 | |
| 26 | GF8 | Which of the following tobacco products do you use daily? | | | | | | | Cigarette | | | |  | | | | 1 | |
|  |  |  |  |  |  |  |  |  | Hand roll cigarette | | | |  | | | | 2 | |
|  |  |  |  |  |  |  |  |  | Pipes | | | |  | | | | 3 | |
|  |  |  |  |  |  |  |  |  | Cigars | | | |  | | | | 4 | |
|  |  |  |  |  |  |  |  |  | Others | | | |  | | | | 5 | |
| 27 | GF9 | Do you use a snuff? | | | | | | | Yes | | | | | | | | 1 | |
|  |  |  |  |  |  |  |  |  | No | | | | | | | | 2 | |
| 28 | LM1 | Are you near a regular smoker at home or work? | | | | | | | Yes | | | | | | | | 1 | |
|  |  |  |  |  |  |  |  |  | No | | | | | | | | 2 | |
| **3.2 Menstrual status** | | | | | | | | | | | | | | | | | | |
| 29 | LM2 | Are your periods regular? | | Regular | | | | | | | | | | | | | 1 | |
|  |  |  |  | Irregular | | | | | | | | | | | | | 2 | |
|  |  |  |  | Menopause | | | | | | | | | | | | | 3 | |
| 30 | LM3 | How many days of bleeding do you usually have each period? | |  | | | | | | | | | | | |  | | |
| 1. **Skincare** | | | | | | | | | | | | | | | | | | |
| 31 | LM4 | Do you clean your face every night? | | Yes | | | | | | | | | | | | 1 | | |
|  |  |  |  | No | | | | | | | | | | | | 2 | | |
|  |  |  |  | Often | | | | | | | | | | | | 3 | | |
| 32 | LM5 | What kind of cleaner do you usually use to clean your face? | | By water | | | | | | | | | | | | 1 | | |
|  |  |  |  | Cleansing foam | | | | | | | | | | | | 2 | | |
|  |  |  |  | Cleansing milk or cleansing cream | | | | | | | | | | | | 3 | | |
|  |  |  |  | Liquids containing alcohol | | | | | | | | | | | | 4 | | |
|  |  |  |  | Use all of these | | | | | | | | | | | | 5 | | |
| 36 | LM6 | Do you use sunscreen cream when you go outside? | | Always | | | | | | | | | | | | 1 | | |
|  |  |  |  | Often | | | | | | | | | | | | 2 | | |
|  |  |  |  | Never | | | | | | | | | | | | | 3 | |
| 37 | LM8 | Do you get a professional skin care? | | Always | | | | | | | | | | | | | 1 | |
|  |  |  |  | Often | | | | | | | | | | | | | 2 | |
|  |  |  |  | Never | | | | | | | | | | | | | 3 | |
| 38 | LM9 | How often do you get a professional skin care? | | Every week | | | | | | | | | | | | | 1 | |
|  |  |  |  | 1-2 times in a month | | | | | | | | | | | | | 2 | |
|  |  |  |  | 1-2 times in a season | | | | | | | | | | | | | 3 | |
|  |  |  |  | 1-2 times in a year | | | | | | | | | | | | | 4 | |
| 39 | PR1 | How many years have you been getting a professional skin care? | | Up to 1 year | | | | | | | | | | | | | 1 | |
|  |  |  |  | 1-5 years | | | | | | | | | | | | | 2 | |
|  |  |  |  | 6-9 years | | | | | | | | | | | | | 3 | |
|  |  |  |  | 10 years or more | | | | | | | | | | | | | 4 | |
| 40 | PR2 | How well do you think you take care of your face? | | Very well | | | | | | | | | | | | | 1 | |
|  |  |  |  | Good | | | | | | | | | | | | | 2 | |
|  |  |  |  | Not good | | | | | | | | | | | | | 3 | |
|  |  |  |  | Bad | | | | | | | | | | | | | 4 | |
| 1. **Clinical examination** | | | | | | | | | | | | | | | | | | |
| Physiological parameters | | | | | | | | | | | | | | | | | | |
| 1 | Weight | | Weight /kg/ | |  | | | | | | | | | | | | | |
| 2 | Height | | Height /meter/ | |  | | | | | | | | | | | | | |
| 3 | BMI | | Body mass index | |  | | | | | | | | | | | | | |
| 4 | BP | | Blood pressure | | Right-arm | | | | | |  | | | | | | | |
|  |  |  |  |  | Left-arm | | | | | |  | | | | | | | |
| 5 | Pulse | | Pulse rate in one minute | |  | | | | | | | | | | | | | |
| 6 | Btime | | Breathing time (seconds) | |  | | | | | | | | | | | | | |
| 7 | BBT | | Body balance time (seconds) | |  | | | | | | | | | | | | | |
| 8 | MC | | Mental status (by points) | |  | | | | | | | | | | | | | |
| 9 | ST | | Skin type | | Normal | | | | | | | | | | 1 | | | |
|  |  |  |  |  | Dry | | | | | | | | | | 2 | | | |
|  |  |  |  |  | Oily | | | | | | | | | | 3 | | | |
|  |  |  |  |  | Combination skin | | | | | | | | | | 4 | | | |
| 10 |  | | Skin parameters | | **Moisture (%)** | | | | | | | | | | | | | |
|  |  |  |  |  | 28-34 | | | | | | | | | | 1 | | | |
|  |  |  |  |  | 35-39 | | | | | | | | | | 2 | | | |
|  |  |  |  |  | 40-45 | | | | | | | | | | 3 | | | |
|  |  |  |  |  | **Sebum (number of oil stains per unit S)** | | | | | | | | | | | | | |
|  |  |  |  |  | 300-530 | | | | | | | | | | 1 | | | |
|  |  |  |  |  | 531-650 | | | | | | | | | | 2 | | | |
|  |  |  |  |  | 651-830 | | | | | | | | | | 3 | | | |
|  |  |  |  |  | 831-1000 | | | | | | | | | | 4 | | | |
|  |  |  |  |  | 1001-1200 | | | | | | | | | | 5 | | | |
|  |  |  |  |  | **Wrinkle (μm)** | | | | | | | | | | | | | |
|  |  |  |  |  | 1 | | | | | | | | | | 1 | | | |
|  |  |  |  |  | 2 | | | | | | | | | | 2 | | | |
|  |  |  |  |  | 3 | | | | | | | | | | 3 | | | |
|  |  |  |  |  | 4 | | | | | | | | | | 4 | | | |
|  |  |  |  |  | 5 | | | | | | | | | | 5 | | | |
|  |  |  |  |  | **Pore (μm)** | | | | | | | | | | | | | |
|  |  |  |  |  | 0-2 | | | | | | | | | | 1 | | | |
|  |  |  |  |  | 3-4 | | | | | | | | | | 2 | | | |
|  |  |  |  |  | 5-6 | | | | | | | | | | 3 | | | |
|  |  |  |  |  | 7-8 | | | | | | | | | | 4 | | | |
|  |  |  |  |  | **Pigmentation (Level)** | | | | | | | | | | | | | |
|  |  |  |  |  | 1А | 01 | 2A | | | 05 | | 3A | | 09 |  | | | |
|  |  |  |  |  | 1B | 02 | 2B | | | 06 | | 3B | | 010 |  | | | |
|  |  |  |  |  | 1C | 03 | 2C | | | 07 | | 3C | | 011 |  | | | |
|  |  |  |  |  | 1D | 04 | 2D | | | 08 | |  | |  |  | | | |
|  |  | |  | | **Elasticity (Level)** | | | | | | | | | | | | | |
|  |  |  |  |  | High | | | | | | | | | | **1** | | | |
|  |  |  |  |  | Medium | | | | | | | | | | **2** | | | |
|  |  |  |  |  | Low | | | | | | | | | | **3** | | | |
| 11 |  | | Skin aging grade | | 1. BG | | | | | | | | | | 1 | | | |
|  |  |  |  |  | 1. BG | | | | | | | | | | 2 | | | |
|  |  |  |  |  | 1. BG | | | | | | | | | | 3 | | | |
|  |  |  |  |  | 1. BG | | | | | | | | | | 4 | | | |
|  |  |  |  |  | 1. BG | | | | | | | | | | 5 | | | |
|  |  |  |  |  | 1. BG | | | | | | | | | | 6 | | | |
| 12 | Mel | | Melatonin (pg/ml) | |  | | | | | | | | | | | | | |
| 13 | EGF | | EGF (ng/l) | |  | | | | | | | | | | | | | |

**QUESTIONNAIRE IN MONGOLIA**

**АСУУМЖ СУДАЛГАА, БОДИТ ҮЗЛЭГИЙН ХУУДАС**

| 1. **Ерөнхий хэсэг** | | | | | | | | | | | | | | | | | | |
| --- | --- | --- | --- | --- | --- | --- | --- | --- | --- | --- | --- | --- | --- | --- | --- | --- | --- | --- |
|  | Код | Асуулт | | | | | | Хариулт | | | | | | | | | | Код |
| 1 | I1 | Хаяг | | | | | |  | | | | | | | | | | |
| 2 | I2 | Асуумж авсан өдөр | | | | | | /_ _ / _ _ _ / _ _ _ _ /  өдөр сар жил | | | | | | | | | | |
| 3 | I3 | Холбогдох утасны дугаар | | | | | |  | | | | | | | | | | |
| 1. **Хүн ам зүйн үзүүлэлт** | | | | | | | | | | | | | | | | | | |
| 4 | De1 | Хүйс | | | | | | Эрэгтэй | | | | | | | | | | 1 |
| 5 | De2 |  |  |  |  |  |  | Эмэгтэй | | | | | | | | | | 2 |
| 6 | De3 | Төрсөн он сар өдөр | | | | | | /_ _ / _ _ _ / _ _ _ _ /  өдөр сар жил | | | | | | | | | | |
| 7 | De4 | Нас | | | | | | _ _ _ нас_ _ сар | | | | | | | | | | |
| 8 | De5 | Ам бүлийн тоо | | | | | |  | | | | | | | | | | |
| 9 | De6 | Гэрлэлтийн байдал | | | | | | Гэрлэсэн | | | | | | | | | | 1 |
| 10 | De7 |  |  |  |  |  |  | Салсан | | | | | | | | | | 2 |
| 11 | De8 |  |  |  |  |  |  | Гэрлээгүй | | | | | | | | | | 3 |
| 12 | Di9 | Таны боловсрол | | | | | | Ахлах сургууль | | | | | | | | | | 1 |
|  |  |  |  |  |  |  |  | Коллеж | | | | | | | | | | 2 |
|  |  |  |  |  |  |  |  | Баклавр | | | | | | | | | | 3 |
|  |  |  |  |  |  |  |  | Магистар, Доктор | | | | | | | | | | 4 |
| 13 | SE2 | Та ямар нөхцөлд ажилладаг вэ? | | | | | | Шөнийн ээлжинд | | | | | | | | | | 1 |
|  |  |  |  |  |  |  |  | Гадаа | | | | | | | | | | 2 |
|  |  |  |  |  |  |  |  | Хүнд | | | | | | | | | | 3 |
|  |  |  |  |  |  |  |  | Хортой | | | | | | | | | | 4 |
|  |  |  |  |  |  |  |  | Хэвийн | | | | | | | | | | 5 |
| 15 | SE3 | Та хаана амьдардаг вэ? | | | | | | Хот | | | | | | | | | | 1 |
|  |  |  |  |  |  |  |  | Аймаг | | | | | | | | | | 2 |
|  |  |  |  |  |  |  |  | Сум суурин | | | | | | | | | | 3 |
|  |  |  |  |  |  |  |  | Хөдөө | | | | | | | | | | 4 |
|  |  |  |  |  |  |  |  | Бусад | | | | | | | | | | 5 |
| 16 | SE4 | Та одоогийн оршин суугаа газраа хэр удаан амьдарч байна вэ? | | | | | | | _ _ _ жил | | | | | | | | |  |
| 17 | SE5 | Танайх ямар сууцанд амьдардаг вэ? | | | | | | | Гэр | | | | | | | | | 1 |
|  |  |  |  |  |  |  |  |  | Хашаа байшин | | | | | | | | | 2 |
|  |  |  |  |  |  |  |  |  | Нийтийн байр | | | | | | | | | 3 |
|  |  |  |  |  |  |  |  |  | Орон сууц | | | | | | | | | 4 |
|  |  |  |  |  |  |  |  |  | Бусад | | | | | | | | | 5 |
| 18 | SE6 | Танайх ямар төрлийн усан хангамжийн эх үүсвэрийг унд ахуйн хэрэгцээндээ ашигладаг вэ? | | | | | | | Төвлөрсөн шугаманд холбогдсон халуун, хүйтэн ус | | | | | | | | | 1 |
|  |  |  |  |  |  |  |  |  | Төвлөрсөн шугаманд холбогдсон хүйтэн ус | | | | | | | | | 2 |
|  |  |  |  |  |  |  |  |  | Төвлөрсөн шугаманд холбогдсон ус түгээх цэг | | | | | | | | | 3 |
|  |  |  |  |  |  |  |  |  | Машинаар зөөвөрлөдөг ус | | | | | | | | | 4 |
|  |  |  |  |  |  |  |  |  | Гүний худгийн ус | | | | | | | | | 5 |
|  |  |  |  |  |  |  |  |  | Гар худгийн ус | | | | | | | | | 6 |
|  |  |  |  |  |  |  |  |  | Ил задгай булаг шандны ус | | | | | | | | | 7 |
|  |  |  |  |  |  |  |  |  | Голын ус | | | | | | | | | 8 |
|  |  |  |  |  |  |  |  |  | Цас мөсний ус | | | | | | | | | 9 |
|  |  |  |  |  |  |  |  |  | Бусад | | | | | | | | | 10 |
| 19 | SE7 | Танай өрхийн сарын орлого \төгрөг\ хэд вэ? | | | | | | |  | | | | | | | | | |
| 1. **Амьдралын хэв маяг, зан үйл** | | | | | | | | | | | | | | | | | | |
| 20 | SE8 | Та ямар хоол ихэвчлэн иддэг вэ? | | | | | | | Mахан хоол | | | | | | | | | 1 |
|  |  |  |  |  |  |  |  |  | Цагаан хоол | | | | | | | | | 2 |
| 21 | SE9 | Та зөвхөн цагаан хоол иддэг бол хэр удаан хэрэглэж байна вэ? | | | | | | | 1 жил хүртэл | | | | | | | | | 1 |
|  |  |  |  |  |  |  |  |  | 1-4 жил | | | | | | | | | 2 |
|  |  |  |  |  |  |  |  |  | 5-9 жил | | | | | | | | | 3 |
|  |  |  |  |  |  |  |  |  | 10 буюу түүнээс | | | | | | | | | 4 |
| 22 | GF1 | Та шөнө хэдэн цаг унтдаг вэ? | | | | | | | 4-5 цаг | | | | | | | | | 1 |
|  |  |  |  |  |  |  |  |  | 6-7 цаг | | | | | | | | | 2 |
|  |  |  |  |  |  |  |  |  | 8-10 цаг | | | | | | | | | 3 |
| 23 | GF2 | Зуны улиралд өглөөний 7 цагаас оройн 6 цаг хүртэл наранд ойролцоогоор хэр их удаан байдаг вэ? | | | | | | | .......... цаг | | | | | | | | | |
|  |  |  |  |  |  |  |  |  | ........ минут | | | | | | | | | |
| **3.1 Tамхины хэрэглээ** | | | | | | | | | | | | | | | | | | |
| 24 | GF6 | Та тамхи татдаг уу? | | | | | | | Тийм | | | | | | | | 1 | |
|  |  |  |  |  |  |  |  |  | Үгүй | | | | | | | | 2 | |
| 25 | GF7 | Хэдэн наснаас эхэлж тамхи татсан бэ? | | | | | | | _ _ жил | | | | | | | |  | |
|  |  |  |  |  |  |  |  |  | Мэдэхгүй | | | | | | | | 99 | |
| 26 | GF8 | Өдөрт дунджаар дараах төрлийн тамхинаас хэдэн ширхэг татдаг вэ? | | | | | | | Үйлдвэрийн тамхи | | | |  | | | | 1 | |
|  |  |  |  |  |  |  |  |  | Гараар ороосон тамхи | | | |  | | | | 2 | |
|  |  |  |  |  |  |  |  |  | Гаансан тамхи | | | |  | | | | 3 | |
|  |  |  |  |  |  |  |  |  | Навчин тамхи | | | |  | | | | 4 | |
|  |  |  |  |  |  |  |  |  | Бусад | | | |  | | | | 5 | |
| 27 | GF9 | Та хамрын тамхи татдаг уу? | | | | | | | Тийм | | | | | | | | 1 | |
|  |  |  |  |  |  |  |  |  | Үгүй | | | | | | | | 2 | |
| 28 | LM1 | Танай гэр болон ажил дээр байнга тамхи татдаг хүн байгаа юу? | | | | | | | Тийм | | | | | | | | 1 | |
|  |  |  |  |  |  |  |  |  | Үгүй | | | | | | | | 2 | |
| **3.2 Эмэгтэйчүүдийн асуумж** | | | | | | | | | | | | | | | | | | |
| 29 | LM2 | Таны сарын тэмдэг ирдэг үү? | | Тогтмол ирдэг | | | | | | | | | | | | | 1 | |
|  |  |  |  | Алдагдаж ирдэг | | | | | | | | | | | | | 2 | |
|  |  |  |  | Цэвэршилт | | | | | | | | | | | | | 3 | |
| 30 | LM3 | Таны сарын тэмдэг хэд хоног ирдэг вэ? | |  | | | | | | | | | | | |  | | |
| 1. **Арьс арчилгаа** | | | | | | | | | | | | | | | | | | |
| 31 | LM4 | Та нүүрээ орой бүр цэвэрлэдэг үү? | | Тийм | | | | | | | | | | | | 1 | | |
|  |  |  |  | Үгүй | | | | | | | | | | | | 2 | | |
|  |  |  |  | Хааяа | | | | | | | | | | | | 3 | | |
| 32 | LM5 | Та нүүрний арьсаа цэвэрлэхдээ ихэвчлэн ямар арга хэрэглэдэг вэ? | | Усаар | | | | | | | | | | | | 1 | | |
|  |  |  |  | Цэвэрлэгч савангаар | | | | | | | | | | | | 2 | | |
|  |  |  |  | Сүүн шингэн эсвэл тосон цэвэрлэгч | | | | | | | | | | | | 3 | | |
|  |  |  |  | Спирт агуулсан шингэн | | | | | | | | | | | | 4 | | |
|  |  |  |  | Бүгдийг нь хэрэглэдэг | | | | | | | | | | | | 5 | | |
| 36 | LM6 | Та гадаа гарахдаа нарны хамгаалалтын тос хэрэглэдэг үү? | | Үргэлж | | | | | | | | | | | | 1 | | |
|  |  |  |  | Хааяа | | | | | | | | | | | | 2 | | |
|  |  |  |  | Үгүй | | | | | | | | | | | | | 3 | |
| 37 | LM8 | Та гоо сайхны салонд үйлчлүүлдэг үү? | | Үргэлж | | | | | | | | | | | | | 1 | |
|  |  |  |  | Хааяа | | | | | | | | | | | | | 2 | |
|  |  |  |  | Үгүй | | | | | | | | | | | | | 3 | |
| 38 | LM9 | Та гоо сайхны салонд ямар давтамжтай үйлчлүүлдэг вэ? | | Долоо хоног бүр | | | | | | | | | | | | | 1 | |
|  |  |  |  | Сард 1-2 удаа | | | | | | | | | | | | | 2 | |
|  |  |  |  | Улиралд 1-2 удаа | | | | | | | | | | | | | 3 | |
|  |  |  |  | Жилд 1-2 удаа | | | | | | | | | | | | | 4 | |
| 39 | PR1 | Та гоо сайхны салонд үйлчлүүлээд хэдэн жил болж байна вэ? | | Нэг хүртэлх жил | | | | | | | | | | | | | 1 | |
|  |  |  |  | 1-5 жил | | | | | | | | | | | | | 2 | |
|  |  |  |  | 6-9 жил | | | | | | | | | | | | | 3 | |
|  |  |  |  | 10 ба түүнээс дээш жил | | | | | | | | | | | | | 4 | |
| 40 | PR2 | Та өөрийгөө нүүрний арьсаа хэр сайн арчилдаг гэж үзэж байна вэ? | | Маш сайн | | | | | | | | | | | | | 1 | |
|  |  |  |  | Сайн | | | | | | | | | | | | | 2 | |
|  |  |  |  | Дунд | | | | | | | | | | | | | 3 | |
|  |  |  |  | Муу | | | | | | | | | | | | | 4 | |
| 1. **Бодит үзлэг** | | | | | | | | | | | | | | | | | | |
| Физиологийн үзүүлэлтүүд | | | | | | | | | | | | | | | | | | |
| 1 | Weight | | Биеийн жин /кг/ | |  | | | | | | | | | | | | | |
| 2 | Height | | Биеийн өндөр /meter/ | |  | | | | | | | | | | | | | |
| 3 | BMI | | Биеийн жингийн индекс | |  | | | | | | | | | | | | | |
| 4 | BP | | Артерийн даралт | | Баруун гарт | | | | | |  | | | | | | | |
|  |  |  |  |  | Зүүн гарт | | | | | |  | | | | | | | |
| 5 | Pulse | | Судасны цохилт нэг минутанд | |  | | | | | | | | | | | | | |
| 6 | Btime | | Амьсгал барих хугацаа (сек) | |  | | | | | | | | | | | | | |
| 7 | BBT | | Биеийн тэнцвэр барих хугацаа (сек) | |  | | | | | | | | | | | | | |
| 8 | MC | | Сэтгэхүйн шалгуур (оноогоор) | |  | | | | | | | | | | | | | |
| 9 | ST | | Арьсны хэв шинж | | Хэвийн | | | | | | | | | | 1 | | | |
|  |  |  |  |  | Хуурай | | | | | | | | | | 2 | | | |
|  |  |  |  |  | Тослог | | | | | | | | | | 3 | | | |
|  |  |  |  |  | Холимог | | | | | | | | | | 4 | | | |
| 10 |  | | Арьсны физиологийн үзүүлэлтүүд | | **Чийглэг (%)** | | | | | | | | | | | | | |
|  |  |  |  |  | 28-34 | | | | | | | | | | 1 | | | |
|  |  |  |  |  | 35-39 | | | | | | | | | | 2 | | | |
|  |  |  |  |  | 40-45 | | | | | | | | | | 3 | | | |
|  |  |  |  |  | **Тослог (нэгж S дахь тосон толбын тоо)** | | | | | | | | | | | | | |
|  |  |  |  |  | 300-530 | | | | | | | | | | 1 | | | |
|  |  |  |  |  | 531-650 | | | | | | | | | | 2 | | | |
|  |  |  |  |  | 651-830 | | | | | | | | | | 3 | | | |
|  |  |  |  |  | 831-1000 | | | | | | | | | | 4 | | | |
|  |  |  |  |  | 1001-1200 | | | | | | | | | | 5 | | | |
|  |  |  |  |  | **Үрчлээ (μm)** | | | | | | | | | | | | | |
|  |  |  |  |  | 1 | | | | | | | | | | 1 | | | |
|  |  |  |  |  | 2 | | | | | | | | | | 2 | | | |
|  |  |  |  |  | 3 | | | | | | | | | | 3 | | | |
|  |  |  |  |  | 4 | | | | | | | | | | 4 | | | |
|  |  |  |  |  | 5 | | | | | | | | | | 5 | | | |
|  |  |  |  |  | **Нүхжилт (μm)** | | | | | | | | | | | | | |
|  |  |  |  |  | 0-2 | | | | | | | | | | 1 | | | |
|  |  |  |  |  | 3-4 | | | | | | | | | | 2 | | | |
|  |  |  |  |  | 5-6 | | | | | | | | | | 3 | | | |
|  |  |  |  |  | 7-8 | | | | | | | | | | 4 | | | |
|  |  |  |  |  | **Нөсөөжилт (Зэрэг)** | | | | | | | | | | | | | |
|  |  |  |  |  | 1А | 01 | 2A | | | 05 | | 3A | | 09 |  | | | |
|  |  |  |  |  | 1B | 02 | 2B | | | 06 | | 3B | | 010 |  | | | |
|  |  |  |  |  | 1C | 03 | 2C | | | 07 | | 3C | | 011 |  | | | |
|  |  |  |  |  | 1D | 04 | 2D | | | 08 | |  | |  |  | | | |
|  |  | |  | | **Уян хатан чанар (Зэрэг)** | | | | | | | | | | | | | |
|  |  |  |  |  | High | | | | | | | | | | **1** | | | |
|  |  |  |  |  | Medium | | | | | | | | | | **2** | | | |
|  |  |  |  |  | Low | | | | | | | | | | **3** | | | |
| 11 |  | | Арьсны хөгшрөлтийн зэрэг | | 1. BG | | | | | | | | | | 1 | | | |
|  |  |  |  |  | 1. BG | | | | | | | | | | 2 | | | |
|  |  |  |  |  | 1. BG | | | | | | | | | | 3 | | | |
|  |  |  |  |  | 1. BG | | | | | | | | | | 4 | | | |
|  |  |  |  |  | 1. BG | | | | | | | | | | 5 | | | |
|  |  |  |  |  | 1. BG | | | | | | | | | | 6 | | | |
| 12 | Мel | | Мелатонин (pg/ml) | |  | | | | | | | | | | | | | |
| 13 | EFG | | ЭӨФ (ng/l) | |  | | | | | | | | | | | | | |
